# Animal Welfare Awareness and Career Aspirations Among Undergraduates in Animal Science-Related Disciplines: A Survey in Northeast China

**DOI:** 10.3390/ani16121908

**Published:** 2026-06-19

**Authors:** Xiaodong Zhu, Yihan Hong, Yuhan Yao, Hanqing Sun, Xiang Li

**Affiliations:** 1 College of Animal Science and Technology, Northeast Agricultural University, Harbin 150030, China; 2 College of Biological Science and Food Engineering, Southwest Forestry University, Kunming 650224, China

**Keywords:** higher education, animal welfare, career aspirations, undergraduates, China

## Abstract

For students in animal science-related disciplines, animal welfare education may be associated with their understanding of human–animal relationships and willingness to pursue animal-related careers. However, empirical research from developing countries such as China remains limited. To address this gap, we conducted a survey among undergraduates at Northeast Agricultural University to examine the associations of demographic characteristics, educational exposure, and animal-related experiences with animal welfare awareness and career aspirations. A total of 346 valid responses were obtained. The results showed that students who encountered animal welfare through school demonstrated significantly higher levels of self-reported animal welfare awareness (*p* < 0.001). Binary logistic regression further confirmed that the channel remained significantly associated with the level of awareness (OR = 8.714, *p* < 0.001). Furthermore, gender and pet-keeping experience were significantly associated with career aspirations in both univariate and logistic regression analysis. In addition, although 50.00% of respondents considered primary school to be the optimal stage for animal welfare education, 81.48% of those exposed through school channels reported their first exposure at university. These findings highlight the need for a progressive animal welfare education framework, transitioning from life education and human–animal relationship training at the primary level to professional practice education in higher education. This approach may help students develop more stable and scientifically informed understanding of animal welfare, as well as a stronger sense of professional identity.

## Simple Summary

Students studying animal-related disciplines may be influenced by animal welfare education in terms of both their attitudes towards animals and their willingness to pursue careers in the animal sector. To analyse this influence, this study conducted a questionnaire survey among undergraduates at Northeast Agricultural University. The results indicate that there is a significant correlation between educational channels and perceptions of animal welfare. Gender, experience of pet ownership and career intentions were all found to be significantly correlated. Furthermore, there was a marked discrepancy between the timing of respondents’ actual exposure to animal welfare education and their expectations regarding such exposure. We therefore recommend the establishment of a progressive animal welfare education system. This should involve the implementation of life education and guidance on human–animal relationships at primary school level, alongside the provision of specialised practical courses at higher education level.

## 1. Introduction

The ‘One Health’ approach emphasises the interconnectedness of humans, animals, and the environment and addresses shared health threats such as zoonotic diseases, antimicrobial resistance and food safety [1]. Within this framework, animal welfare, as an important component of the ‘One Health’ concept, has received increasing global attention and has become an important issue for global sustainable development [2,3,4]. Studies have shown that well-implemented animal welfare standards can generate multiple advantages, including restoring ecosystems [5], reducing the risk of zoonotic diseases [6], improving product quality [7,8], and even generating more employment opportunities [9]. However, there are significant differences in animal welfare regulations and standards among different regions and countries. For example, developed regions such as the European Union (EU) have established relatively comprehensive animal welfare regulations [10,11] to advance animal welfare. By contrast, many developing countries face multiple challenges, including a lack of animal welfare legislation and insufficient consumer awareness, and animal welfare issues are often not given sufficient priority [12,13].

China is the world’s largest livestock-producing country and faces significant challenges in farm animal welfare [14]. Over the past few decades, China’s livestock industry has undergone a rapid transition towards large-scale and intensive production [15]. This transition has significantly enhanced the supply capacity of livestock products, whilst also making animal welfare issues more prominent in both production practices and professional training. However, some scholars have pointed out that there are only a few reports on China’s farm animal welfare in the scientific literature [16], which to some extent limits the international academic community’s understanding of animal welfare in China. One possible explanation is that the development of animal welfare in China began relatively late. Since Jun Bao introduced the concept of animal welfare to China in 1994, the field has developed domestically for only about three decades [3]. Although China has not yet enacted comprehensive animal welfare legislation [17], provisions relating to animal welfare are included in laws and regulations governing farm animal husbandry, laboratory animal management and wildlife protection [18,19]. However, these provisions remain fragmented and primarily focus on disease control, product quality, or conservation rather than animal welfare per se, which can only provide a minimum level of protection. The greatest challenge in promoting animal welfare lies in fundamentally transforming public attitudes towards animal welfare. In particular, students in animal science and related disciplines represent a key source of future professionals for the husbandry, research, education, and related sectors [20]. Their perceptions, attitudes and practices may directly or indirectly influence the future development of animal welfare in China [21,22]. Therefore, a systematic evaluation of their welfare awareness and perceptions of specific animal issues is essential for informing educational reform in animal science. Several studies have begun to explore this topic by surveying university students across different educational levels [23], genders [24], and living or animal-related experiences [25]. These studies provide foundational data for understanding students’ basic perceptions of animal welfare. However, some research findings also suggest that the factors influencing attitudes towards animal welfare may be complex and context-dependent. For example, Carnovale et al. [26] found that high school graduates were more concerned about animal welfare than university graduates, suggesting that the relationship between educational level and attitudes towards animal welfare may not be linear. Mariti, Pirrone, Albertini, Gazzano and Diverio [20], on the other hand, reported that familiarity with work in the livestock industry actually reduced the likelihood of veterinary students taking a positive stance on animal welfare. These findings are particularly important for students in animal science-related disciplines, as they receive both specialist education and practical training in animal production, and face career prospects within the animal industry. However, systematic research on animal welfare awareness and career aspirations among undergraduates in animal science-related disciplines remains limited. Conducting such research within the Chinese context is of great significance in supplementing the international body of animal welfare research with evidence from China and for understanding how animal welfare education is associated with the formation of professional identity among students in animal-related disciplines.

Northeast Agricultural University is one of the first universities in China to have undertaken teaching and research in animal welfare. Based on the university’s unique strengths in animal environment and applied animal behaviour science, Jun Bao introduced the concept of animal welfare into the course of Applied Animal behaviour Science since 1994. Subsequently, in 2002, Weiguo Cui incorporated the ‘Animal Welfare’ course into the undergraduate curriculum of the animal science programme. These developments have made the university one of the earliest institutions in China to offer systematic animal welfare education across bachelor’s, master’s, and doctoral programmes. This cohort therefore provides a useful case for examining animal welfare awareness among undergraduates in animal science-related disciplines against the backdrop of the early development of animal welfare education in China. In light of the current state of animal welfare education in China, this study conducted a cross-sectional survey targeting undergraduates at the College of Animal Science and Technology, Northeast Agricultural University. This study examines how the channel and timing of first exposure to animal welfare, along with academic background and personal animal-related experiences, are associated with students‘ self-reported understanding of animal welfare and their willingness to pursue animal-related careers. Through an examination of the issues outlined above, this study aims to identify the key factors associated with perceptions of animal welfare and career aspirations, thereby providing a basis for improving animal welfare education and the training of professionals within China’s animal industry.

## 2. Materials and Methods

### 2.1. Study Design and Participants

This study was a single-institution cross-sectional survey. The study population comprised undergraduate students enrolled at the College of Animal Science and Technology, Northeast Agricultural University. The first-year intake of the college consists of students in Animal Production programmes (approximately 240 students per cohort) and Animal Science programmes (approximately 20 students per cohort, in the integrated Bachelor’s-Master’s-PhD programme). Among these, students in the Animal Production programme receive a general education in their first year and, in their second year, are free to choose one of three specialisations—Animal Science, Aquaculture, or Grassland Science—based on their preferences, with an approximate distribution ratio of 5:2:1. The questionnaire was distributed to a total of 987 undergraduate students in the College of Animal Science and Technology (a total of 437 men and 550 women), and participation was voluntary. A total of 379 questionnaires were returned, representing a response rate of 38.40%. The response rate may be partly attributable to the survey timing (end-of-term and winter break) and the absence of participation incentives. After excluding 33 invalid questionnaires (e.g., due to incorrect year of study, incorrect major, or responses exhibiting obvious logical inconsistencies), 346 valid responses were included in the final analysis.

### 2.2. Questionnaire Design

The main body of the questionnaire comprised 25 closed-ended questions, divided into two main sections: the first section covers basic personal information, such as gender and field of study (Q1–Q8); the second section examines students’ attitudes towards selected animal welfare-related issues and animal-use scenarios included in the questionnaire (Q9–Q25). This study used Q9 as an indicator of respondents’ level of animal welfare awareness. This item reflects respondents’ self-reported familiarity with and understanding of the concept of animal welfare, rather than a score based on an objective knowledge test. Some respondents may overestimate or underestimate their own level of understanding. A self-assessment indicator was used for practical reasons and because the study focused on subjective perceptions. However, this limitation should be considered when interpreting the results. Future research could incorporate objective knowledge tests to assess levels of animal welfare awareness more comprehensively.

Prior to the formal survey, the questionnaire was pre-tested by two PhD students and five master’s students to assess the clarity of the questions, the accuracy of the response options, the use of scientific terminology, and the overall flow of the survey. Certain wording and response options based on the pre-test feedback to produce the final questionnaire. The complete questionnaire and all response options are shown in Supplementary Questionnaire S1.

### 2.3. Data Collection and Ethical Approval

The questionnaire was distributed through a web link and QR code shared via WeChat, and respondents could complete it voluntarily using a mobile phone, computer, or tablet. The questionnaire took approximately 10 min to complete. All questionnaire items were mandatory; therefore, there were no missing data for the main variables among valid responses. No incentives were offered for participation to avoid influencing students’ voluntary participation. Consequently, students with a greater interest in animal welfare or other animal-related issues may be more likely to complete the questionnaire, this factor should be considered when assessing the representativeness of the sample. As a result, the findings may overestimate students’ awareness of animal welfare and underestimate the proportion of students with unclear career intentions, because students with lower interest in the topic may have been less likely to participate.

The survey was approved by the Ethics Committee of Northeast Agricultural University (Approval No.: NEAUEC20250252). Key information regarding ethical risks and privacy protection was provided in the introductory section of the survey. Furthermore, the purpose of student participation was emphasised, and it was made clear that all responses would remain anonymous. All data will be used solely for statistical analysis within this study and will be presented in aggregated form.

### 2.4. Statistical Analysis

All statistical analyses were conducted in R. Prior to conducting between-group comparisons, descriptive statistics were performed on the questionnaire responses from the entire sample. Differences between groups were assessed using the chi-square test. For consistency with the subsequent logistic regression analyses, Q9 and Q10 were dichotomised in the same manner as in the regression models before the chi-square analyses were performed. All statistical tests were two-tailed, and a *p* < 0.05 was considered statistically significant.

Furthermore, to analyse the factors associated with animal welfare awareness and the intention to pursue animal-related careers, this study fitted multivariated logistic regression models. Before model construction, multicollinearity was assessed among the seven independent variables (Q1–Q7), all of which were categorical. To avoid model instability, variables with strong collinearity or conceptual overlap were not entered into the main model simultaneously.

First, we used the level of animal welfare awareness (Q9) as the dependent variable in Model 1. In the regression analysis, we further dichotomised Q9. The response option ‘Heard & fully understand’ was defined as indicating a higher level of self-assessed animal welfare awareness and assigned a value of 1. As the sample size for ‘Never heard of’ was small, the response options ‘Heard & partially understand’ and ‘Never heard of’ were combined into a category representing non-high self-assessed awareness of animal welfare, assigned a value of 0. Model 2 used career intention (Q10) as the dependent variable. As the sample size for ‘Unwilling’ was small, ‘Willing’ was assigned a value of 1 and defined as a clear intention to pursue a career in the field. The response options ‘Unwilling’ and ‘It doesn’t matter’ were combined and assigned a value of 0, defined as an unclear intention to pursue a career in this field. Given the cross-sectional design, this study focused on associations rather than causal effects.

In the chi-square analysis, Cramer’s V was used as a measure of effect size to assess the strength of association between categorical variables; in the logistic regression analysis, the odds ratios (OR), 95% confidence interval (CI) and events-per-variable (EPV) were used to reflect the strength and precision of the association between variables. The statistical power of the chi-square tests and logistic regression analyses was also estimated.

## 3. Results

### 3.1. Respondent Characteristics and Differences Between Groups

The questionnaire first examined the respondents’ basic characteristics (Q1–Q6). The respondent group consisted predominantly of women (65.03%), with the majority being students of animal science (90.17%). Most respondents reported animal-related life experiences: 72.83% had lived in rural areas, 61.85% had experience rearing farm animals, and 67.34% had experience keeping pets.

Regarding animal welfare-related content (Q7–Q11), 189 respondents (54.62%) indicated that they had been exposed to animal welfare through school channels, such as courses or lectures, with 81.48% of these students stating that their first exposure to the subject occurred during their university studies. Furthermore, only 13.87% of respondents stated that they had never heard of animal welfare, and the majority expressed a desire to pursue animal-related careers in the future (78.32%). Finally, nearly half (50.00%) of respondents believed that primary school was the most appropriate stage for animal welfare education, whilst only 6.94% considered university to be the most suitable stage. Full responses are available in Supplementary Table S1.

To investigate differences in students’ perceptions of animal welfare and their career aspirations across different background characteristics, this study conducted a univariate analysis. The results of the chi-square test revealed significant differences in the self-reported animal welfare awareness among respondents from different year groups, disciplines and exposure channels. Respondents who had been exposed to animal welfare through school channels were significantly more likely to select ‘Heard & fully understand’ (68.78% vs. 20.38%), with the difference being statistically significant (χ^2^ = 80.689, *p* < 0.001, Cramer’s V = 0.483, power > 0.999). There were also significant differences in the self-reported animal welfare awareness across different year groups and majors (*p* < 0.001). The descriptive distribution results further indicate that the proportion of first- and second-year students with an awareness of animal welfare is relatively low, at 22.76% and 9.09% respectively; from the third year onwards, this proportion rises sharply to 65.03%, with fourth-year students showing the highest proportion at 80.85%. In other words, undergraduate students’ awareness of animal welfare increases significantly once they reach their third year. The specific results are shown in Table 1.

Regarding willingness to pursue a career in the field, gender, year group and pet ownership experience showed significant associations in univariate analysis. Notably, male respondents exhibited a lower willingness to pursue a career than female respondents (71.90% vs. 81.78%), a difference that was significant (χ^2^ = 4.521, *p* = 0.033, Cramer’s V = 0.114, Power = 0.566). Respondents with pet-owning experience expressed a higher willingness to pursue a career in this field than those without such experience (84.55% vs. 65.49%), a difference that was highly significant (χ^2^ = 16.287, *p* < 0.001, Cramer’s V = 0.217, Power = 0.981). No significant association was observed between exposure to animal-related subjects in school education and the willingness to pursue a career in this field (χ^2^ = 1.116, *p* = 0.291, Cramer’s V = 0.057, Power = 0.184). The remaining results are shown in Table 2.

### 3.2. Logistic Regression Analysis of the Relationship Between Levels of Awareness of Animal Welfare and Career Aspirations

Prior to conducting logistic regression, we first assessed the multicollinearity among the seven candidate explanatory variables. The results indicated a strong correlation between the year of study (Q2) and the channels of exposure to animal welfare (Q7); these two variables may jointly reflect the extent of students’ exposure to relevant coursework during their professional training. Furthermore, due to the limitations of the cross-sectional design, this study was unable to fully distinguish the effects of other potential factors influenced by the year group variable (such as professional knowledge, personal experience, and changes in professional identity). Therefore, to avoid model instability, this study retained only the exposure channel variables directly relevant to the research question in the main model and did not include the year group variable. Nonetheless, the univariate analysis (Table 1 and Table 2) already revealed a significant association between year of study and animal welfare awareness. Future research could employ a longitudinal design to further distinguish the relationships between year group, course exposure and perceptions of animal welfare.

To further analyse the factors associated with higher self-reported animal welfare awareness among students, we constructed a binary logistic regression model (as shown in Table 3). We first calculated the EPV values for the models. The EPV value for Model 1 was 27, indicating that the model had a sufficient number of events relative to the number of predictor variables included. The results indicate that, after controlling for other factors, the route of exposure remained significantly associated with the self-reported animal welfare awareness (OR = 8.714, *p* < 0.001, Power > 0.999). Furthermore, after controlling for the route of exposure, there was a significant association between the student’s field of study and animal welfare awareness (*p* = 0.001). Students majoring in Animal Science demonstrated a higher relative advantage in achieving higher welfare awareness. However, due to the small sample sizes for Aquaculture and Grassland Science, these findings should be interpreted with caution. The remaining main results are presented in Table 3.

A second binary logistic regression model was fitted with ‘Willingness for Animal-Related Future Work’ as the dependent variable. The EPV value for Model 2 is 12.5, indicating that the model has a sufficient number of events relative to the number of predictor variables included. The results indicate that pet-owning experience is significantly associated with the willingness to pursue animal-related careers. Students with pet-owning experience had a significantly higher relative likelihood of pursuing animal-related work than those without such experience (OR = 2.795, *p* < 0.001, Power = 0.971). Similarly, after controlling for other variables, gender remained significantly associated with the willingness to pursue animal-related careers, with male respondents exhibiting a lower relative likelihood of pursuing such careers (OR = 0.535, *p* = 0.027, Power = 0.598). The remaining main results are presented in Table 4.

### 3.3. Analysis of Respondents’ Attitudes Towards the Welfare of Specific Animals

This study also examined respondents’ attitudes towards the welfare of different types of animals (laboratory animals, farm animals, companion animals and wildlife). Given that experience of keeping pets was found to be significantly associated with the intention to pursue animal-related careers, this study describes the distribution of respondents’ attitudes towards welfare scenarios relating to companion animals.

**Figure 1:**
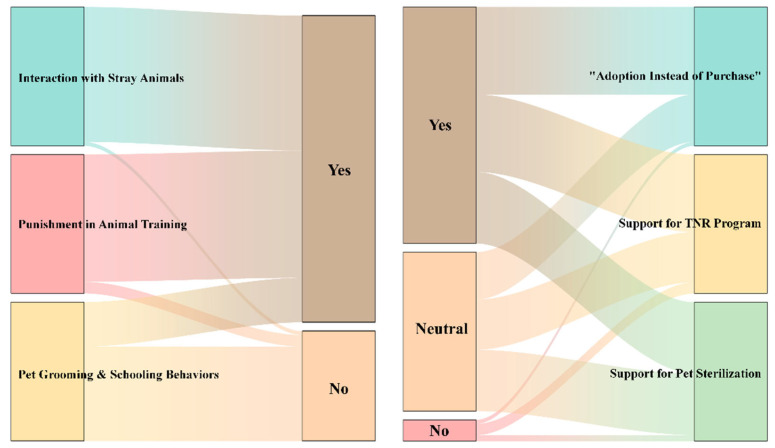
Respondents’ responses towards companion animal-related questions (*n* = 346). The width of each streamline in the figure is proportional to the number of respondents who chose the corresponding answer option and has no relation to causal pathways or temporal sequences. The six questions are as follows: Have you interacted with stray animals? [Q12, Yes (336, 97.11%), No (10, 2.89%)]; Do you accept the use of punishment measures in animal training? [Q13, Yes (317, 91.62%), No (29, 8.38%)]; Do you support dressing pets in clothes or sending them to pet schools for socialisation? [Q14, Yes (111, 32.08%), No (235, 67.92%)]; Do you support the “adoption instead of purchase”? [Q15, Yes (218, 63.01%), Neutral (118, 34.10%), No (10, 2.89%)]; Do you support the Trap-Neuter-Return (TNR) model? [Q16, Yes (193, 55.78%), Neutral (125, 36.13%), No (28, 8.09%)]; Do you support the sterilization of pets? [Q17, Yes (178, 51.45%), Neutral (153, 44.22%), No (15, 4.34%)].

With regard to companion animals (Q12–17), Figure 1 provides a visual summary of respondents’ answers to six companion animal-related questions. The left panel presents responses to binary questions regarding interaction with stray animals, acceptance of punitive training, and pet dressing or training-school behaviours, while the right panel presents responses to three-option questions regarding adoption instead of purchase, TNR, and pet sterilization. Specifically, almost all respondents (97.11%) reported interacting with stray animals (including frequent interaction, occasional interaction and almost no interaction). The vast majority of respondents (91.62%) accepted, or accepted as a last resort, the use of punishment in pet training, and 32.08% of respondents approved of practices such as dressing pets in clothes or sending them to pet schools for training. Furthermore, the majority of students supported ‘adoption over buying’ (63.01%) and the ‘Trap-Neuter-Return (TNR)’ model for stray animals (55.78%). Finally, the proportion of respondents supporting pet neutering (51.45%) and those remaining neutral (44.22%) far exceeded the proportion opposed (4.34%).

In addition, we conducted a chi-square analysis of respondents’ awareness of animal welfare and their willingness to work in the field, based on their answers to questions regarding companion animals. However, due to the significant disparity in the number of respondents for the different options, we have not included this analysis in this experiment; please refer to Supplementary Table S2 for the specific results.

**Figure 2:**
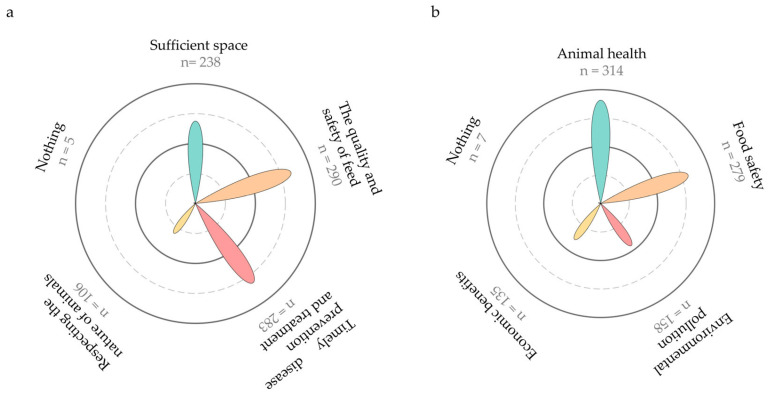
Distribution of respondents’ responses to farm animal-related questions (*n* = 346). (**a**) During the process of raising farm animals, which of the following aspects are relatively important? (Q18, multiple choice question, total response frequency: 922); (**b**) Which of the following aspects will mainly be affected by the welfare of farm animals? (Q19, multiple choice question, total response frequency: 893).

Although farm animal breeding experience was not significantly associated with self-reported animal welfare awareness or willingness to pursue animal-related future work, we have nevertheless presented respondents’ views on farm animal welfare, given that the survey participants were students in animal science-related disciplines. For farm animals (Q18–19, Figure 2), ensuring ‘The quality and safety of feed’ and ‘Timely disease prevention and treatment’ were considered the most important factors (Figure 2a). Students perceived farm animal welfare as primarily affecting ‘Animal Health’, followed by ‘Food Safety’ (Figure 2b).

## 4. Discussion

The study indicates that there are certain discrepancies between the level of self-reported awareness of animal welfare among undergraduate students in animal science-related disciplines and the pathways through which their career intentions are formed. Given the single-institution, voluntary, and cross-sectional design of this study, the findings should be interpreted as associations rather than causal relationships. Higher education is a key avenue through which students learn about animal welfare, yet there is a marked discrepancy between this and the timing of exposure they would ideally expect. In contrast, students’ career intentions are more closely linked to personal experiences, particularly those relating to pet ownership. Gender also plays a significant role.

### 4.1. University Education and Animal Welfare Awareness

The study found that more than half of the respondents were first exposed to animal welfare through school, and that school-based exposure was significantly associated with higher self-reported awareness of animal welfare. In China, higher education remains a key setting in which students in animal science-related disciplines systematically learn about animal welfare. Furthermore, among the 189 respondents who learned about animal welfare through school channels, more than 80% stated that they were first introduced to animal welfare-related knowledge during their undergraduate studies. Interestingly, however, nearly half of the respondents believed that primary school was the optimal stage for animal welfare education, while only 6.94% considered university to be the best stage. Thus, university-level education appears to be the main channel through which many students report being introduced to animal welfare in a more structured way, although this does not align with the stage that many respondents considered ideal. This discrepancy may have important implications for how students conceptualise animal welfare. When students first encounter animal welfare at university, they often learn about it within professional contexts such as production, slaughter and processing, or laboratory animal management. This may lead some students to view animal welfare primarily as a technical production standard or ethical constraint, rather than as a broader value linked to personal life, social responsibility, and professional ethics. By contrast, if concepts of animal welfare are gradually constructed from the primary and secondary education stages through life education and education on human–animal relationships, students are more likely to develop internalised and stable awareness and values. Although this study cannot directly verify this hypothesis, future research could design longitudinal tracking studies or compare cohorts first exposed to animal welfare education at different ages to examine the long-term impact of the timing of education on cognitive structures and professional identity.

Unlike other livestock-related courses, animal welfare has the dual attributes of scientific knowledge and values education. On the one hand, animal welfare education at university level plays an irreplaceable role for students in animal science and veterinary-related disciplines. A previous survey of US undergraduates revealed that the majority of respondents felt that animal welfare courses were beneficial to their future careers [22]. University courses can help students systematically understand the scientific foundations of animal welfare, assessment methods and practical applications in production, thereby integrating animal welfare principles with the development of professional competencies. In this study, respondents who were exposed to animal welfare through university courses reported higher self-reported awareness of animal welfare, suggesting a positive correlation between specialised courses, university education and students’ perceived familiarity with animal welfare-related concepts. However, due to the limitations of the cross-sectional design, self-selection bias and prior interest in animal welfare cannot be ruled out. Students who were already interested in animal welfare may have been more likely to remember, value, or actively seek out school-based exposure to animal welfare-related content. On the other hand, animal welfare also encompasses value-based aspects such as respect for life, empathy and the relationship between humans and animals. For example, Hazel et al. [27] found that animal welfare education at university can positively enhance veterinary students’ attitudes towards animals. However, exposure to these value-based aspects need not be delayed until higher education. Previous research has shown that childhood is a critical period for the development of empathy and complex moral cognition [28]. Therefore, introducing animal welfare education at an earlier stage could enhance its role in early life education and the shaping of values. More importantly, this would facilitate the wider dissemination of animal welfare concepts among the general public.

Previous scholars have noted that most Chinese primary and secondary school textbooks portray animals as friends of humans, emphasising their natural ecological attributes [29]. Whilst such portrayals may help foster an initial emotional connection to animal conservation and harmonious coexistence with nature, they are insufficient to help students understand the multifaceted roles animals play in human society. This may limit students’ ability to develop a comprehensive understanding of animal welfare, particularly because explicit exposure to the concept of animal welfare remains limited at that stage. Compared to the gradual formation of stable value perceptions during early development, students who only begin to systematically engage with animal welfare at university level are often confronted not merely with the principles of animal welfare themselves, but also with the specific contradictions and limitations inherent in real-world production, animal management and professional practice. In this context, whilst animal welfare education may help to enhance students’ knowledge and ethical standards, it may also lead them to view animal welfare as inextricably linked to practical production, rather than as a continuous framework that permeates personal life, social responsibility and professional ethics.

These findings suggest the potential value of considering animal welfare education as a progressive system. Such an approach may respond to the preference expressed by nearly half of the respondents for animal welfare education to be introduced at an earlier stage, while also preserving the role of in-depth education at university level for students in animal husbandry-related disciplines by linking animal welfare to practical production. For example, at the primary and secondary education stage, life education and nature education could be used to guide students towards understanding that animals have basic needs and the capacity to feel, thereby fostering an initial awareness of respect for life and the humane treatment of animals. A prime example is the CFL (Caring for Life Education) programme launched by ACTAsia (Action for Compassion, Together). This programme aims to help Asian children aged 6 to 12 develop compassion and a sense of responsibility towards animals, people and the environment. Several studies have examined such programmes, and the findings indicate that they are effective in increasing lower elementary students’ prosociality [30]. At the secondary school level, the complex nature of human–animal relationships can be further explored. For instance, by examining the different roles animals play—such as agricultural products, companion animals, or laboratory subjects—students can gradually come to understand that animal welfare encompasses a variety of social contexts, including farm animals and laboratory animals. At the higher education level, animal welfare education could be further integrated with production practices and career guidance, helping students to understand animal welfare principles whilst recognising practical pathways for their application in real-world production and professional practice [31]. This progressive educational approach, ranging from early values education to specialised university-level study, may better support students in developing a more stable, informed, and practice-oriented understanding of animal welfare, although its effects require further longitudinal or intervention-based evaluation. It is worth noting that a study by Carnovale, Xiao, Shi, Arney and Phillips [26] found that high school graduates were more concerned about animal welfare than university graduates, which appears to differ from the finding of this study that university education was associated with higher self-reported awareness. Possible explanations include the fact that the subjects of this study were animal science students, for whom animal welfare content may be integrated throughout their university curriculum, whereas this is not necessarily the case in general higher education; furthermore, emotion-driven cognition at the high school level and knowledge-driven cognition at the university level may follow different pathways. Future longitudinal studies could track changes in animal welfare-related knowledge and attitudes towards animals among the same cohort of students from secondary school through to university, in order to better examine the temporal relationships between educational stage and students’ animal welfare-related perceptions.

### 4.2. Career Aspirations of Undergraduates in Animal Science-Related Disciplines

Compared with the target research group, the proportion of female respondents in this sample was relatively high. This may indicate that female students are more willing to participate in surveys concerning animal welfare and career aspirations in animal-related fields, or it may reflect their greater interest in this topic. In this study, a statistically significant association was found between gender and career aspirations, with male respondents reporting a lower willingness to pursue animal-related careers. Further binary logistic regression analysis revealed that, even after adjustment for other variables, gender remained significantly associated with career aspirations. Animal-related careers encompass a wide range of career paths. On the one hand, they include roles closely linked to production practices, such as livestock farming, slaughter and processing, and laboratory animal management. On the other hand, they also encompass fields such as companion animal care, animal welfare and veterinary medicine, which place greater emphasis on care, communication and ethical responsibility. It should be noted that different students may have varying interpretations of what constitutes an ‘animal-related career’. Occupations have long been shaped by gender stereotyping. Livestock farming and agriculture have tended to be closely associated with men [32], resulting in the contributions of women in these sectors being underestimated [33,34]. In addition, an increasing number of women have entered animal care professions such as veterinary medicine in recent years [35,36]. In some veterinary schools, the proportion of female students has even reached 70% to 75% [37]. Some studies on veterinary medicine have also found that women are less willing to choose veterinary work related to livestock [38]. Therefore, the findings of this study do not necessarily imply that women are better suited to animal-related professions, nor that men lack compassion for animals. Rather, they may reflect gendered perceptions of animal-related careers among students. Future research could consider explicitly listing specific career paths, such as farm animals, companion animals or wildlife, to more accurately measure and validate gendered perceptions of different professions among undergraduate students in animal science.

These findings offer valuable insights for the training of professionals in animal-related fields. When delivering vocational education, higher education institutions can help students recognise the diversity of careers in this sector; by broadening their understanding of the animal industry, this may assist students of all genders in developing a sense of professional identity within the field.

### 4.3. Companion Animal Contact, Career Aspirations, and Animal Welfare Awareness

Companion animal-related responses provide an important lens through which to examine the relationship between emotional attachment to animals and self-reported animal welfare awareness. We observed that experience of keeping pets was significantly correlated with the respondents’ willingness to pursue a career in this field, consistent with previous research [25]. Respondents with pet-keeping experience were more willing to pursue animal-related careers, suggesting that daily contact with animals and emotional bonds may be associated with career aspirations. However, the cross-sectional design of this study does not allow us to determine whether pet-keeping experience contributes to career aspirations, or whether students who are already interested in animals are more likely to keep pets.

It should be noted that whilst experience of keeping pets may be associated with a more positive attitude towards animal welfare, this does not necessarily mean that the individual possesses a scientific and comprehensive understanding of animal welfare. Existing research has also found that pet owners tend to treat animals with greater equality and kindness, whilst also being more willing to purchase livestock and poultry products that meet high animal welfare standards [25,39,40]. However, in this study, over 90% of respondents accepted, or accepted as a last resort, the use of punitive measures during pet training, and one-third of students endorsed behaviours such as dressing pets in clothes or shoes, or bringing them to school for socialisation. Such behaviours indicate that people expect pets to conform to human aesthetic standards and behavioural patterns, constituting a subtle form of objectification [41,42]. On the one hand, people wish for pets to possess their own freedom; on the other, they demand absolute obedience to humans [43]. This positioning presupposes the objectification of companion animals from the outset, creating a fundamental conflict with the respondents’ assertion in this survey that pets should not be treated as commodities. In addition, The vast majority of respondents in this survey have interacted with stray animals—a finding consistent with a previous survey conducted in China—this indicates that the Chinese public generally harbours a fondness for companion animals [44]. However, instances of abuse and commercial exploitation of companion animals do still occur [44], and the abandonment of companion animals remains a widespread global issue [45]. This further illustrates the consequences of viewing pets solely from the perspective of human needs and convenience. Furthermore, more than half of the respondents supported the sterilisation of both pets and stray animals. In other words, the majority of respondents were willing to sacrifice the animal’s autonomy in exchange for more manageable behaviour and lower social costs. This constitutes an objectifying intervention implemented by humans to facilitate the better integration of companion animals into human society. It is worth noting that a very high proportion of respondents adopted a neutral stance on the issue of neutering. This may reflect a sense of indecision among respondents. Although they are aware that neutering is a common means of controlling pet populations and preventing disease, they remain concerned about ethical controversies and potential health risks [46,47], hence their neutral stance. It is clear, therefore, that students’ attitudes towards companion animals encompass not only considerations of animal welfare, but are also intertwined with subjective views centred on human needs, with an emphasis on convenience, control and social perceptions.

Consequently, how to transform this emotionally based affinity into a scientific and rational understanding of animal welfare is a key issue that requires attention in the context of companion animal engagement. From an educational perspective, higher education institutions should place greater emphasis on the pedagogical value of meaningful contact with animals. In addition to traditional courses and professional practice, universities can provide students with experiential learning opportunities through companion animal care placements, animal behaviour observation, campus animal welfare activities or related voluntary work. By combining students’ existing emotional experiences with animal behaviour and animal welfare science, this approach may help students progress from simply liking animals to understanding them, thereby fostering a more rational, stable and practical understanding of animal welfare. It must be acknowledged that the data on attitudes towards companion animals in this study only describe students’ support for or opposition to specific practices (such as punitive measures, dressing animals, and neutering), without directly measuring whether students are aware of the potential conflict between these behaviours and animal welfare. For example, future studies could examine how students reconcile support for ‘adoption over purchasing’ with acceptance of punitive training measures. Future research could further explore the internal tensions within students’ attitudes through situational comparisons or in-depth interviews.

### 4.4. Limitations of the Study and Future Directions

This study provides preliminary evidence for understanding the animal welfare awareness and career aspirations among undergraduate students in animal science-related disciplines in China. As the study was conducted via a fully voluntary online survey with no incentives offered, there may be some self-selection bias in the sample. Students with an interest in animal welfare or animal-related careers may have been more inclined to participate in this survey, leading to an overestimation of the overall level of animal welfare awareness and career aspirations. Furthermore, the respondents were drawn from universities in China that have been engaged in animal welfare teaching and research for a relatively long time; they may have received more education or academic exposure related to animal welfare. Additionally, this study used self-reported familiarity as a measure of animal welfare awareness rather than an objective knowledge assessment. Self-reported results may differ from students’ actual knowledge levels. Therefore, caution should be exercised when generalising the findings of this study to groups from other universities, disciplines, or educational stages.

Furthermore, as this study employs a cross-sectional design, it can only reveal correlations between variables and cannot infer causal relationships. To ensure model stability, the study categorised the level of animal welfare awareness into two groups by merging ‘Never heard of’ and ‘Heard & partially understand’, which may result in some loss of information. The former category indicate a complete lack of basic familiarity with animal welfare concepts, while the latter suggests at least some prior exposure to relevant concepts and a certain level of foundational understanding. Consequently, the regression results of this study are best interpreted as an analysis of the differences between ‘high self-assessed awareness’ and ‘non-high self-assessed awareness’, rather than a complete representation of a continuous gradient of animal welfare awareness.

Future research could adopt a longitudinal design to track changes in animal welfare awareness and career intentions among the same cohort of students across different stages of their academic journey. Furthermore, ordered logistic regression, multi-class logistic regression or objective knowledge assessments could be employed to analyse differences between varying levels of awareness in greater depth. Additionally, the sample scope could be expanded to include students from different types and levels of higher education institutions, as well as those with diverse academic backgrounds, in order to validate the applicability of this study’s findings and to further explore the potential development of a phased, progressive animal welfare education system. Furthermore, this study did not distinguish further between the types of future employment sought by the respondents; future research could incorporate more detailed occupational categories in order to gain a more accurate understanding of students’ career interests and career planning. Such research would help transform the widespread sentiment of animal protection within society into a more scientific, stable and practice-oriented understanding of animal welfare.

## 5. Conclusions

Our findings indicate that higher education is the primary avenue through which undergraduates in animal science-related disciplines systematically learn about animal welfare. However, 50.00% of respondents believed that such education should begin as early as primary school. There is a positive correlation between university education and self-reported animal welfare awareness, while career aspirations are more closely linked to personal experiences, particularly those involving pet ownership. It is worth noting that this study employs a cross-sectional design and therefore cannot establish causal relationships. In summary, the findings of this study indicate that the development of a tiered, progressive animal welfare education system holds practical value; this serves as an educational insight from this study and a direction for future research. Primary and secondary schools can provide education on the relationship between life and animals, whilst universities should offer courses in animal welfare science, practical production and career guidance. Such a progressive approach may help foster a more stable, rational and practically oriented understanding of animal welfare. In the future, longitudinal or intervention studies could be conducted to further evaluate the effectiveness of animal welfare education at different educational levels.

## Figures and Tables

**Table 1:** Chi-square analysis of factors associated with animal welfare awareness.

	Q9. Awareness of Animal Welfare			
	χ^2^	*p*	V	Power
Gender	3.822	0.051	0.105	0.498
Grade	88.369	<0.001	0.505	>0.999
Animal Science Major	12.889	<0.001	0.193	0.949
Rural Living Experience	0.524	0.469	0.039	0.112
Animal Breeding Experience	0.032	0.859	0.010	0.054
Pet-keeping Experience	0.043	0.835	0.011	0.055
Animal welfare education via school	80.689	<0.001	0.483	>0.999

Note: χ^2^ = Chi-square statistic; *p* = *p*-value; V = Cramer’s V; Power = Statistical Power. Significant associations are indicated by *p* < 0.05.

**Table 2:** Chi-square analysis of factors associated with Willingness for Animal-Related Work.

	Q10. Willingness for Animal-Related Future Work			
	χ^2^	*p*	V	Power
Gender	4.521	0.033	0.114	0.566
Grade	9.408	0.024	0.165	0.732
Animal Science Major	1.329	0.249	0.062	0.211
Rural Living Experience	0.593	0.441	0.041	0.120
Animal Breeding Experience	2.094	0.148	0.078	0.304
Pet-keeping Experience	16.287	<0.001	0.217	0.981
Animal welfare education via school	1.116	0.291	0.057	0.184

Note: χ^2^ = Chi-square statistic; *p* = *p*-value; V = Cramer’s V; Power = Statistical Power. Significant associations are indicated by *p* < 0.05.

**Table 3:** Binary logistic regression analysis of awareness of animal welfare.

	B	Sig.	OR	95% CI		Power
				Lower	Upper	
Gender	−0.349	0.196	0.705	0.415	1.197	0.258
Animal Science Major	1.608	0.001	4.995	1.853	13.465	0.953
Rural Living Experience	−0.170	0.553	0.844	0.482	1.478	0.102
Animal Breeding Experience	0.088	0.744	1.092	0.645	1.850	0.065
Pet-keeping Experience	0.007	0.978	1.007	0.592	1.713	0.043
Animal welfare education via school	2.165	<0.001	8.714	5.247	14.472	>0.999
Constant	−2.661	<0.001	0.070			

Note: B = regression coefficient; Sig. = significance level (*p*-value); OR = odds ratio; 95% CI = 95% confidence interval for OR; Power = Statistical Power. Sig. < 0.05 was considered statistically significant.

**Table 4:** Binary logistic regression analysis of employment intentions.

	B	Sig.	OR	95% CI		Power
				Lower	Upper	
Gender	−0.626	0.027	0.535	0.307	0.930	0.598
Animal Science Major	0.432	0.305	1.541	0.675	3.519	0.185
Rural Living Experience	0.138	0.647	1.148	0.636	2.071	0.074
Animal Breeding Experience	0.340	0.234	1.405	0.802	2.461	0.227
Pet-keeping Experience	1.028	<0.001	2.795	1.631	4.789	0.971
Animal welfare education via school	−0.401	0.150	0.670	0.388	1.156	0.286
Constant	0.444	0.387	1.559			

Note: B = regression coefficient; Sig. = significance level (*p*-value); OR = odds ratio; 95% CI = 95% confidence interval for OR; Power = Statistical Power. Sig. < 0.05 was considered statistically significant.

## Data Availability

None of the data were deposited in an official repository. The raw data supporting the conclusions of this article will be made available by the authors on request.

## Supplementary Materials

The following supporting information can be downloaded at: https://www.mdpi.com/article/10.3390/ani16121908/s1, Supplementary Questionnaire S1: The complete questionnaire and options. Supplementary Table S1: Demographic characteristics and corresponding key issues. Supplementary Table S2: Chi-square test based on questions regarding companion animals.
