# Supplementary material for: Animal Welfare Awareness and Career Aspirations Among Undergraduates in Animal Science-Related Disciplines: A Survey in Northeast China
Source: Animals (Basel). 2026 Jun 19;16(12):1908. doi: 10.3390/ani16121908 (PMC13295797; doi:10.3390/ani16121908)
Supplement: Supplementary file 1 [file animals-16-01908-s001.zip › animals-4376268-supplementary/Supplementary Questionnaire S1.pdf]

1. Gender: Male (1), Female (2)

2. Grade: First year (1), Second year (2), Third year (3), Fourth year (4)

3. Major: Animal Science (1), Aquaculture/Grassland Science (2)

4. Do you have rural living experience? Yes (1), No (2)

5. Do you have experience in raising farm animals? Yes (1), No (2)

6. Do you have experience in keeping pets? Yes (1), No (2)

7. Did you first get exposed to animal welfare through school channels? Yes (1), No (2)

8. At which educational stage did you first learn about animal welfare? Preschool & Primary School (1), Middle School (2), University (3)

9. How well do you know about animal welfare? Heard & fully understand (1), Heard & partially understand (2), Never heard of (3)

10. Would you like to engage in animal-related work in the future? Willing (1), Unwilling (2), It doesn’t matter (3)

11. To establish a scientific concept of human-animal coexistence, when do you think animal welfare education should be carried out? Preschool (ages 0 - 6) (1), Primary School (ages 6 - 12) (2), Middle School (ages 12 - 18) (3), University (after the age of 18) (4), Not needed (5)

12. Have you interacted with stray animals: Yes (1), No (2)

13. Do you accept the use of punishment measures in animal training: Yes (1), No (2)

14. Do you support dressing pets in clothes or sending them to pet schools for socialisation?: Yes (1), No (2)

15. Do you support the “adoption instead of purchase”: Yes (1), Neutral (2), No (3)

16. Do you support the Trap-Neuter-Return (TNR) model: Yes (1), Neutral (2), No (3)

17. Do you support the sterilization of pets: Yes (1), Neutral (2), No (3)
