# Supplementary material for: Animal Welfare Awareness and Career Aspirations Among Undergraduates in Animal Science-Related Disciplines: A Survey in Northeast China
Source: Animals (Basel). 2026 Jun 19;16(12):1908. doi: 10.3390/ani16121908 (PMC13295797; doi:10.3390/ani16121908)
Supplement: Supplementary file 1 [file animals-16-01908-s001.zip › animals-4376268-supplementary/Supplementary Table S1.pdf]

Table S1. Demographic characteristics and corresponding key issues

Question	Answer	n	%
Q1. Gender	Male	121	34.97
	Female	225	65.03
Q2. Grade	First year	123	35.55
	Second year	33	9.54
	Third year	143	41.33
	Fourth year	47	13.58
Q3. Major	Animal science	312	90.17
	Aquaculture/Grassland science	34	9.83
Q4. Rural Living Experience	Yes	252	72.83
	No	94	27.17
Q5. Animal Breeding Experience	Yes	214	61.85
	No	132	38.15
Q6. Pet – keeping Experience	Yes	233	67.34
	No	113	32.66
Q7. Animal welfare education via school	Yes	189	54.62
	No	157	45.38
Q8. First Education Stage for Animal Welfare Learning	Preschool & primary school	3	1.59
	Middle school	32	16.93
	University	154	81.84
Q9. Awareness of Animal Welfare	Heard & fully understand	162	46.82
	Heard & partially understand	136	39.31
	Never heard of	48	13.87
Q10. Willingness for Animal - Related Future Work	Willing	271	78.32
	Unwilling	15	4.34
	It doesn't matter	60	17.34
Q11. Optimal Timing for Animal Welfare Education	Preschool (ages 0 - 6)	60	17.34
	Primary school (ages 6 - 12)	173	50.00
	Middle school (ages 12 - 18)	87	25.14
	University (after the age of 18)	24	6.94
	Not needed	2	0.58

Note: n = number of respondents; % = percentage of respondents.
