# Supplementary material for: Animal Welfare Awareness and Career Aspirations Among Undergraduates in Animal Science-Related Disciplines: A Survey in Northeast China
Source: Animals (Basel). 2026 Jun 19;16(12):1908. doi: 10.3390/ani16121908 (PMC13295797; doi:10.3390/ani16121908)
Supplement: Supplementary file 1 [file animals-16-01908-s001.zip › animals-4376268-supplementary/Supplementary Table S2.pdf]

Table S2. Chi-square test based on questions regarding companion animals

	Q9. Awareness of Animal Welfare		Q10. Willingness for Animal-Related Future Work	
	χ^2	p	χ^2	p
Interaction with Stray Animals	2.270	0.322	6.240	0.044
Punishment in Animal Training	8.026	0.018	1.853	0.396
Pet Grooming & Schooling Behaviours	1.503	0.472	0.736	0.692
"Adoption Instead of Purchase"	7.764	0.101	9.784	0.044
Support for TNR Programme	0.269	0.992	1.399	0.844
Support for pet Sterilization	0.093	0.999	3.360	0.500

Note: χ^2 = Chi-square statistic; p = p-value. Significant associations are indicated by $p < 0.05$.
